# A Novel Antibody Humanization Method Based on Epitopes Scanning and Molecular Dynamics Simulation

**DOI:** 10.1371/journal.pone.0080636

**Published:** 2013-11-21

**Authors:** Ding Zhang, Cai-Feng Chen, Bin-Bin Zhao, Lu-Lu Gong, Wen-Jing Jin, Jing-Jun Liu, Jing-Fei Wang, Tian-Tian Wang, Xiao-Hui Yuan, You-Wen He

**Affiliations:** 1 MOH Key Laboratory of Systems Biology of Pathogens, Institute of Pathogen Biology, Chinese Academy of Medical Sciences & Peking Union Medical College, Beijing, China; 2 Harbin Veterinary Research Institute, Harbin, Chinese Academy of Agricultural Sciences, Harbin, China; 3 Department of Immunology, Duke University Medical Center, Durham, North Carolina, United States of America; 4 Department of Anesthesiology, The General Hospital of Chinese People's Armed Police Forces, Beijing, China; Institut Pasteur, France

## Abstract

1-17-2 is a rat anti-human DEC-205 monoclonal antibody that induces internalization and delivers antigen to dendritic cells (DCs). The potentially clinical application of this antibody is limited by its murine origin. Traditional humanization method such as complementarity determining regions (CDRs) graft often leads to a decreased or even lost affinity. Here we have developed a novel antibody humanization method based on computer modeling and bioinformatics analysis. First, we used homology modeling technology to build the precise model of Fab. A novel epitope scanning algorithm was designed to identify antigenic residues in the framework regions (FRs) that need to be mutated to human counterpart in the humanization process. Then virtual mutation and molecular dynamics (MD) simulation were used to assess the conformational impact imposed by all the mutations. By comparing the root-mean-square deviations (RMSDs) of CDRs, we found five key residues whose mutations would destroy the original conformation of CDRs. These residues need to be back-mutated to rescue the antibody binding affinity. Finally we constructed the antibodies in vitro and compared their binding affinity by flow cytometry and surface plasmon resonance (SPR) assay. The binding affinity of the refined humanized antibody was similar to that of the original rat antibody. Our results have established a novel method based on epitopes scanning and MD simulation for antibody humanization.

## Introduction

Monoclonal antibody (mAb) has become promising therapeutics for many diseases, including infection, cancer, and immune disorder diseases [1]. The number of approved mAb therapeutics has grown dramatically. To date, a total of 34 mAbs have been approved in either Europe or the United States for clinical use [2]. The C-type lectin receptor DEC-205 expressed on dendritic cells (DCs) recognizes foreign antigen and induces internalization [3]–[5]. DEC-205 antibody specifically targets antigen to DCs. In vivo experiment showed that use of anti-DEC-205 antibody increases the efficiency of antigen presentation of DCs by ∼1000 fold [6]. Thus, anti-DEC-205 antibody represents an attractive therapeutic mAb candidate. We generated a rat-anti-human DEC-205 antibody 1-17-2 by standard hybridoma technology. The antibody is potent in inducing internalization by DCs. To make use of this antibody for future human application, the antibody needs to be humanized to reduce xeno response [7].

Many methods have been used in antibody humanization [8]–[10]. The early approach is making chimeric antibody [11] that connects variable regions of mouse antibody to the conserved regions of human antibody. The chimeric antibody preserves the antibody binding affinity and specificity well. However, it contains many murine residues in the variable regions that could still induce human anti-murine response [12]. In order to increase the degree of the murine antibody humanization, grafts of CDRs of murine antibody were inserted in a human FRs template [13], [14]. Currently, CDR graft is the basic method in antibody humanization. Many modifications have been made based on CDR graft [15], [16]. However, CDR grafted antibody usually exhibits a decreased or lost binding affinity. Certain key residues in FRs play an important role in holding the conformation of the binding domain. After grafting, the human template may not support the CDRs well in its original conformation, which may cause the alternation of its binding affinity [17], [18].

Another method for humanization is antibody resurfacing, which was first described by Padlan [19]. They substituted the murine residues on the domain surface with their human counterparts to avoid immunogenicity caused by those accessible residues on the surface. The resurfaced antibody reserves the CDRs conformation well, thus maintaining the antibody binding affinity[20]. However, some murine residues inside the domain may increase the risk of being recognized by the host [21].

Precisely docking between the antibody CDRs and antigen is the core characteristics of antibody binding [22]. Conformation of CDRs matched with its original FRs represents the best conformation for the binding [23], [24]. Residue changes within the FRs may impact the CDRs conformation. Though most positions within the FRs may have a relatively slight influence, residue changes in certain positions may drastically alter the CDRs conformation. These key residues play an important role in maintaining the original CDRs conformation. In humanization process, these key residues must be retained to preserve the antibody binding affinity. However, identifying these key positions is a difficult task. The process in identifying these key residues by experiment is hugely time- and labor-consuming.

To overcome these problems, we used two strategies. First, we designed a novel epitope scanning algorithm to identify antigenic residues in rat FRs. By eliminate antigenic amino acids, much less residues in FRs are changed during the first humanization step. Second, we used virtual mutations [25] and MD simulation to study the influence on CDRs structure imposed by the humanization mutations [26]. Mutant and parental CDRs structures were compared, RMSD [27] values were calculated. We found that 5 amino acids on FRs of 1-17-2 were key residues in maintaining the natural CDRs conformation. MD simulation guided our calculation for searching the most reasonable conformation after mutations. Importantly, we have confirmed the validity of our humanization strategy by mutation experiments.

## Materials and Methods

### Cloning of antibody variable regions

Hybridoma 1-17-2 was generated by immunization of rats with hDEC-205 expressing YB2/0 cells (DEC-1-YB2/0) using standard hybridoma technology. Animal use was approved by the Ethics Committee of the Institute of Pathogen Biology of the Chinese Academy of Medical Sciences. Immunized rats were euthanized by CO2 inhalation followed by cervical dislocation. Total RNA was extracted from 10^7^ hybridomas using RNAprep pure Micro Kit (TIANGEN) and reverse transcribed by Superscript® III first-strand Synthesis System using oligo-dT primers (Invitrogen). The cDNA was then poly-G tailed using Terminal Deoxynucleotidyl Dransferase (Promega). Gene amplifications were conducted using Phusion® High-Fidelity DNA Polymerase (NEB). Anchor primer is 5′-CG TCGATGAGCTCTAGAATTCGCATGTGCAAGTCCGATGGTC CCCCCCCCCCCCC -3′. Gene specific primer for light chain is 5′- AGGATGATGTC TTATGAACAA -3′. Gene specific primer for heavy chain is 5′- TCACATTGAGCTT GCTGTA -3′. The PCR products were inserted to pGEM®-T vector (Promega) and sequenced (SinoGenoMax). The VL and VH were analyzed using IMGT database [28]. Six CDRs were defined using Kabat, Chothia and IMGT numbering schemes.

### Fab homology modeling

The 3D structure of 1-17-2 Fab was built by homology modeling method [29] using life science software Discovery Studio 2.5 (Accelrys Software). Web blast tool was used to search templates in the PDB (http://www.rcsb.org). Templates for light, heavy and light-heavy dimmer were searched independently. The templates with the best similarity are under the following PDB_ID: 1ZAN, 2ADG and 1IGY. The sequences of Fab and corresponding templates were aligned accurately. Initial Fab model was built using the Modeller module [30] according to the sequence alignment. An *ab initio* loop prediction algorithm LOOPER [31] was performed to refine CDR3 region in the light and heavy chain. Finally, the energy minimizations (EM) were performed in CHARMM27 forcefield [32] by a 500-step steepest-descent (SD) minimization followed by conjugate gradient (CG) [29] minimization until the final convergence was lower than 0.4184 kJ/(mol·nm) [33], [34].

The final Fab structure was tested on stereo-chemical accuracy with the Procheck [35] online program. Residue compatibility was assessed by the Profile-3D [36] program in Discovery Studio. All the calculations were performed on a Dell PowerEdge 2900 workstation.

### Multiple Sequence Alignment (MSA)

NCBI_Balstp tool was performed in Genbank using the 1-17-2 VL and VH variable region amino acid sequences respectively to search the human templates with the high similarity with the 1-17-2 variable region, incomplete or wrong sequences were removed manually. For light and heavy chain the highest 20 sequence were downloaded under the following accession numbers: Human IgG_VL: AAV40711, AAZ09177, CAJ75520, AAV40708, AAV40705, CAA84388, ABO90609, AAK94864, AAF35180, AAX11221, AAZ09103, ADU32652, AAB68785, AAY33483, CAB51292, CAG27042, AAC08335, ABI74045, ABU90626, ADU32621; Human IgG_VH: AAQ05462, CAD88745, ABI74270, ADX65676, AAC50999, CAB44863, AAQ05399, ABI74197, AAO35730, AAR06599, AAQ87978, ACR16069, ABA26139, ABC67107, AAC09115, ACS95994, CAB37174, ABI74417, ACR16083, AAM75842. MSAs were performed with Clustal W 1.83 [37] software using a progressive algorithm and adjusted manually.

### Epitopes scanning

The epitopes scanning algorithm includes linear epitopes scanning and conformational epitopes scanning. Linear epitopes scanning: Taking every six polypeptide segment within FRs as a probe, short-blast calculation was performed in the aligned sequences. The probe moves forward one amino acid every cycle. Peptide segments that do not have a matching in any human antibody sequence are defined as possible liner epitope, and the rat unique residues were identified [34], . Conformational epitopes scanning: All the residues in FRs were analyzed on the 1-17-2 Fab model built previously. Every two residues were used as a unit. A unit with the space distance of constituent atoms less than 5 angstroms, and not appeared in any human sequences, is defined as a conformational epitope [34]. The epitope scanning calculation was performed in programs written by the Python script language on Dell PowerEdge 2900 workstation.

### Virtual Mutation and MD simulation of Fab

Virtual Mutation (VM) refers to the replacement of a single or multiple amino acids in the atomic 3D model of the molecule [25] using the modeling tool Modeller [30]. The nature of VM is the same with homology modeling. 30 mutants were built using the Modeller module in Discovery Studio 2.5 platform. The energy minimizations were performed as described in Fab homology modeling method.

All the MD simulations were performed with the fast MD simulation software package Gromacs [39] using charmm27 force field [32]. First, the initial model was dissolved in rectangular boxes containing SPC/E (simple-point-charge) [40] water molecules, and a certain number of neutralizing Na+ or Cl- ions were added to neutralize the negative charge. After removing bad contacts energy minimizations and relaxing water solvents by position-restrained MD simulations, a final 5-ns production MD simulation for each Fab was performed under periodic boundary conditions with time step of 2 fs at 310 K (∼37°C). Electrostatic interactions were calculated using the Particle Mesh Ewald (PME) [41] summation scheme. The conformations were stored every 5 ps. Finally, the MD simulation was analyzed in terms of potential energy (PE) and RMSD [42] from the initial model structure to determine whether the structures were balanced using the Gromacs suite of programs. The average structures were selected from the last stable phase and further refined to obtain the final structure. Molecular graphics images were generated using Discovery Studio software. All of the Gromacs MD simulations were performed in the DAWNING Supercomputer Center (64 Cores).

### CDRs superimpose and RMSD calculation

All resulting mutant structures were superimposed with the parent 1-17-2 Fab structure. Pair-wise structural RMSD calculations were performed in Discovery Studio 2.5 platform. The RMSD is the measure of similarity of three-dimensional structures after optimal rigid body superposition. RMSD of the Cα atomic coordinates is calculated using the following formula [42]:
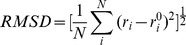



The *r_i_* and *r_i_^0^* are position value of atoms. *N* is the sum of atoms.

### Expression of recombinant antibody

We constructed the chimeric antibody, humanized antibody and refined antibody and expressed the antibodies in vitro using MH and MK vectors (kindly provided by Dr. Linqi Zhang at Tsinghua University, Beijing, China). Human constant regions of H and L chain are contained in the MH and MK vectors respectively. Murine or modified VH and VL were inserted into the open reading frame. The humanized VL and VH fragments were synthesized according to the humanized sequences. Refined VL and VH were generated by introducing site-directed mutations based on humanized VL and VH fragments. The two vectors were co-transfected into HEK 293T cells by lipofectamine 2000 (Invitrogen). Recombinant antibodies were collected from the culture supernatants. Antibodies were concentrated by amicon ultra-centrifugation (Millipore) and purified by HiTrap Protein G affinity column chromatography (GE Healthcare). Labeling of antibody was conducted by APEX Antibody Labeling Kit (Invitrogen). The isotype of rat antibody was determined by SBA Clonotyping System/Beads Rat Isotyping Panel (SouthernBiotech, Birmingham, AL).

### Flow cytometry assay

The antibodies were added to 5×10^5^ YB2/0 cells or DEC-1-YB2/0 cells and incubated at 37°C for 45 min. Cells were washed with PBS twice and then incubated with anti-rat or anti-human antibodies (CW Biotech) for 30 min. After washing, the cells were analyzed by FACSCanto II. For endocytosis assay, 10^6^ DCs were incubated with antibody at 37°C or 4°C for 60 min. After washing with PBS, the cells were fixed by 4% paraformaldehyde (PFA) or permeabilized with Fixation and Permeabilization solution (BD). After fixed or permeabilized, the cells were then stained with APC-labeled anti-CD11c antibodies (BD Bioscience) and incubated for 30 min at 4°C. Cells were resuspended in 1% PFA and applied to FACSCanto II.

### SPR assay

SPR assay was performed on BIACORE T200 instrument with CM5 sensor chips (GE Healthcare). Murine antibodies were immobilized by amine couple kit (GE Healthcare). DEC-205 protein solutions from 500 to 15.625 nM were passed through. Each injection set for 240 sec at the flow rate of 30 µl/min and waited 600 sec for dissociation. At the end of each cycle, Glycine-HCL (PH 2.5) was injected for regeneration. Chimeric and humanized antibodies were measured using human antibody capture kit (GE Healthcare). Anti-Human IgG (Fc) antibody was immobilized. Different antibodies were injected for 60 sec at the flow rate of 10 µl/min. Then DEC-S solution was passed through. At the end of each cycle, 3 M magnesium chloride was injected for regeneration. The sensograms were analyzed by BIAevaluation 3.0 package (GE Healthcare). Equilibrium constant KDs (defined as the kd/ka ratio) were calculated.

### Induction of DCs from PBMCs

Peripheral blood mononuclear cells (PBMCs) were separated from human periphery blood using Ficoll-Paque™ PLUS (GE Healthcare) density gradient centrifugation. The human blood was obtained from healthy donors with approval from the Ethics Committee of Institute of Pathogen Biology after written informed consent was signed. CD14^+^ cells were selected from PBMCs using MACS® Cell Separation system (Miltenyi Biotec). 1×10^8^ PBMCs were mixed with 200 µl of MACS CD14 MicroBeads and incubated for 15 min at 4°C. The cells were then applied to a MS column and washed. The CD14^+^ cells were eluted from the column. DCs were induced from CD14^+^ cells at the condition of 500 U/ml GM-CSF plus 500 U/ml IL-4 for 6 days [43].

### Internalization by human DCs and microscopic imaging

DCs were harvested and washed using phosphate buffered saline (PBS) containing 2% FBS. 1×10^6^ DCs were suspended in 100μl PBS and incubated with 10 µg FITC-labeled DEC-205 antibody at 37°C or 4°C for 60 min. After washing with PBS, APC-labeled anti-CD11c antibodies (BD Bioscience) were added into the cell suspension. After incubation for 30 min at 4°C, 1% paraformaldehyde PFA was used to suspend the cells. The cells were then put in a glass bottom dish and observed in Leica TCS SP5 laser confocal microscope. The lasers used to excite FITC and APC are Argon and HeNe laser respectively.

## Results

### Antibody sequences and the structure of 1-17-2 Fab

**Figure 1:**
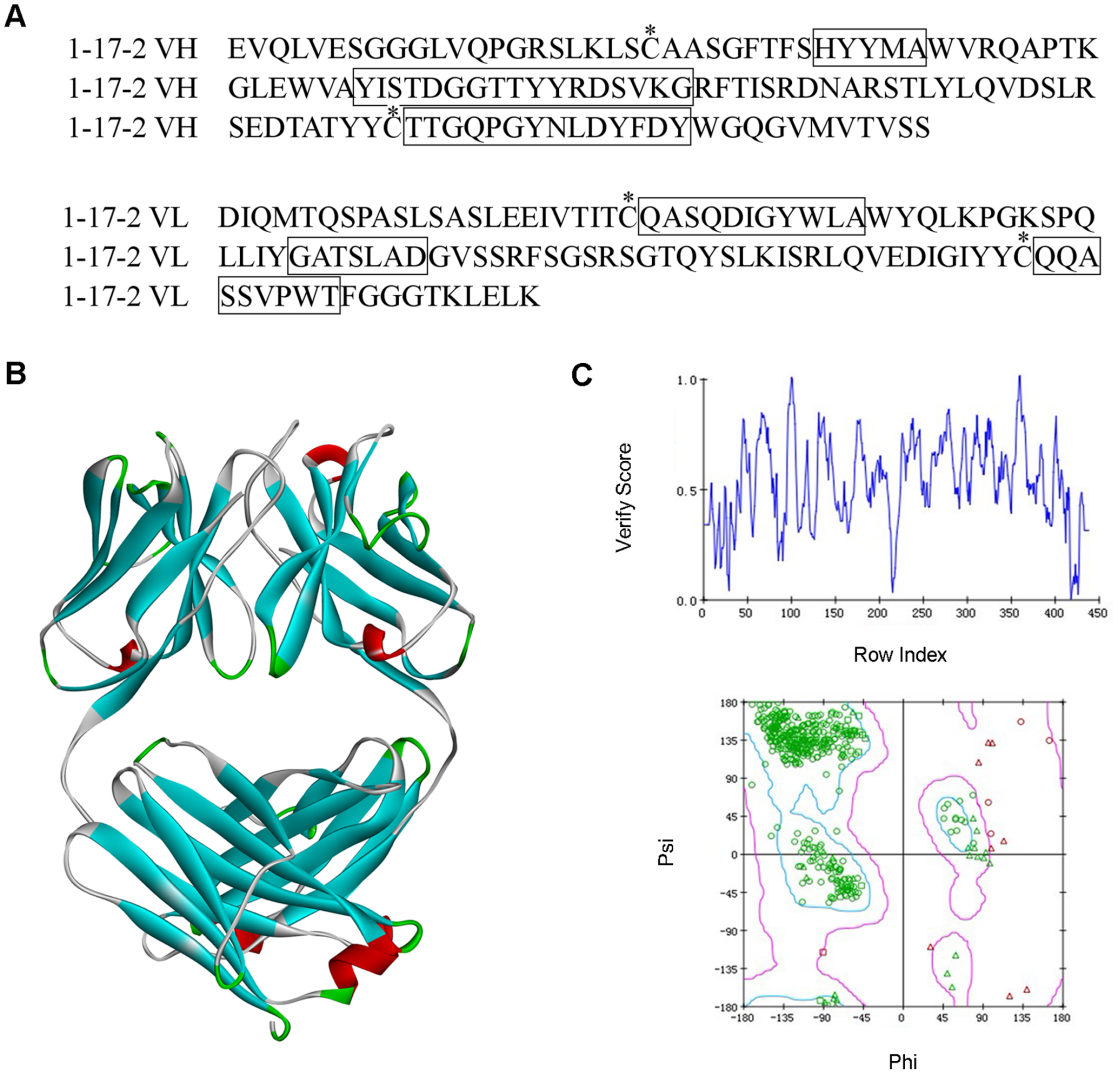
Sequences analysis of antibody 1-17-2. (A, B) Amino acid sequences of 1-17-2. Shown are variable regions of heavy (A) and light (B) chain of 1-17-2. The residues in boxes are CDRs as defined by IMGT. The residues with a star above are the characteristic cysteine residues. (C) 3D model of 1-17-2 Fab. The secondary structures random coil, β sheet, α helix and turn were colored in white, cyan, red and green color respectively. (D) The Profile-3D verification of 1-17-2. Score > 0.0 indicates that the residue is compatible. (E) Ramachandran plots of 1-17-2 Fab structure. The Ramachandran plot shows phi-psi torsion angles of all residues in the structure. Green spots indicate reasonable residues. Red spots indicate un-trusted residues.

We cloned the variable regions of the antibody heavy and light chain by 5′-RACE [44]. Sequence analysis shows that the antibody isotypes are IgG1 and κ respectively, which is consistent with flow cytometric analysis. Cysteine residues in L23, L88, L134, L194 and H22, H96, H148, H203 can form 4 disulfide bonds within the domain. CDRs were identified using the principles of Kabat, Chothia and IMGT schemes. The CDRs and FRs within the variable regions are shown in Figure 1A and 1B. We then used Homology Modeling to predict the 3D structure of 1-17-2 Fab. CDRs are often diverse especially in CDR3 on heavy chain (H-CDR3), so LOOPER program was performed to refine the CDRs conformation. LOOPER uses methods based on de novo physics that reconstruct the loop, ignoring its starting conformation. Thus it breaks the bias inherited from the initial template. The antibody 1-17-2 Fab structure forms stable light-heavy dimer, mainly through hydrophobic interaction (Fig. 1C). Four disulfide bonds were found within the domain. Six CDRs on the top form the antigen-binding surface (Fig. 1C).

Profiles-3D evaluates the compatibility of all amino acids on the 3D structure especially on a hypothetical protein structure. As shown in Figure 1D, all the residues had received a positive verify score, indicating that the primary sequence is compatible on the Fab structure. The Procheck program was employed to evaluate 3D chemical parameters. More than 95% of the main chain φ and ψ dihedral angles in each model were in the core area and only 1% was in the untrusted zone, indicating that the predicted models have good stereochemical features (Fig. 1E).

### Humanization substitutions by Epitopes scanning

**Figure 2:**
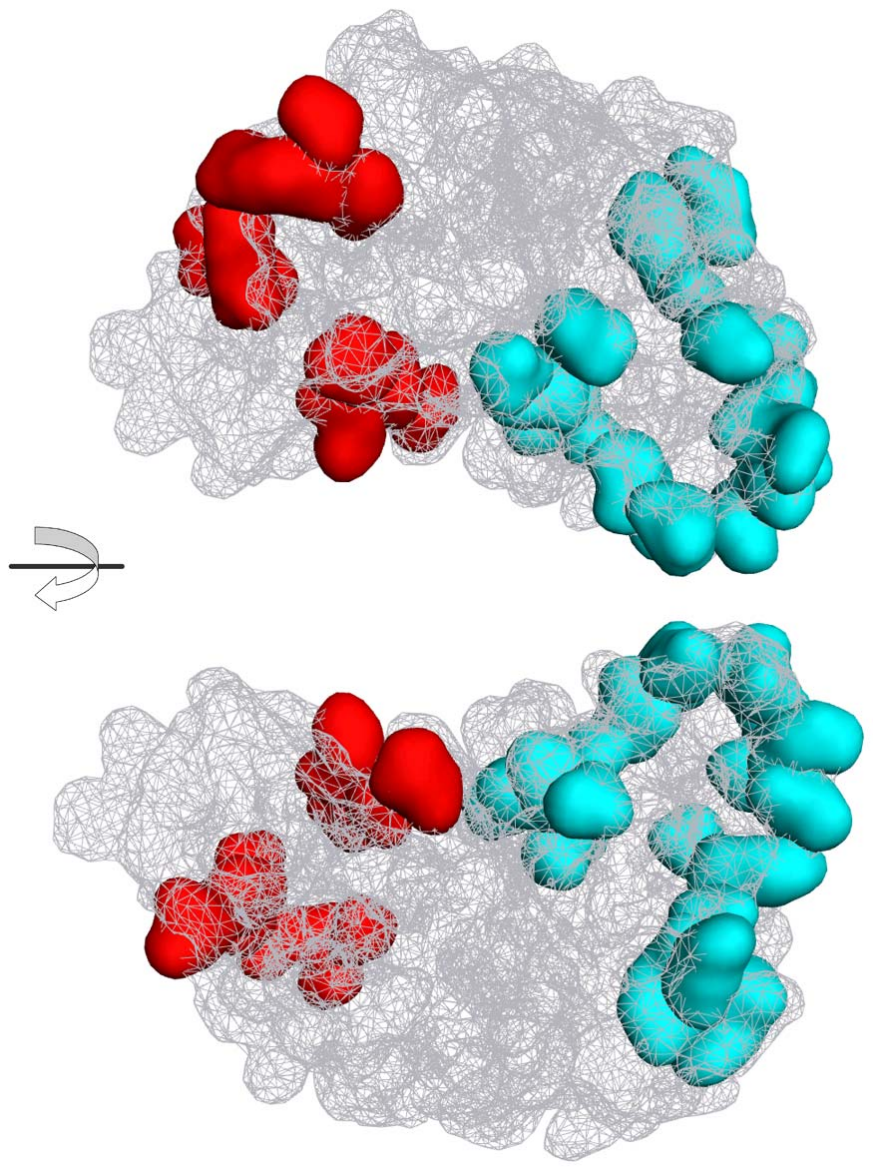
Mapping of the critical 30 amino acids on 1-17-2 Fab model. Two models are shown in opposite view. The surface of Fab was shown in wire mesh. 30 amino acids were shown in solid surface. Amino acids in the heavy chain or light chain are shown in red and cyan respectively.

The first step in humanization is to substitute rat unique amino acids with human source. According to multiple sequence alignments, we found that many residues in FRs are strictly conserved across species. However, in some positions, residues are identical in human sequences but different in rat. These are the rat unique amino acids. In some positions, residues have diversity within human sequences. In these positions, the uniqueness of rat amino acid depends on whether it appears in any human sequence, as well as the circumstance within the radius of 6 residues. We designed an epitope scanning algorithm to find all rat unique amino acids in 1-17-2. In epitope scanning algorithm, we also consider the space distance within two residues that are unique to rat. If the two residues are less than 5 angstroms, they might be close enough to form an conformational epitope. The conformational epitope scanning was used to exclude such instance. As shown Table 1 and 2, 20 residues in light chain and 10 residues in heavy chain were identified as rat unique amino acids. All 30 amino acids were mapped to the 3D structure of 1-17-2 Fab model and most of the residues are located on the surface of the domain (Fig. 2). In the primary humanized Fab, we substituted rat unique residues with the human counterpart in those sites..

**Table 1:** Amino acid mutations in the light chain of the 1-17-2 mAb.

Position	Rat AA	Human AA
9	A	S
15	L	V
16	E	G
17	E	D
18	I	R
38	L	Q
43	S	A
45	Q	K
59	S	P
66	R	G
70	Q	D
71	Y	F
72	S	T
74	K	T
77	R	S
80	V	P
83	I	F
84	G	A
85	I	T
106	L	I

**Table 2:** Amino acid mutations in the heavy chain of 1-17-2 mAb.

Position	Rat AA	Human AA
19	K	R
42	T	G
75	A	S
76	R	K
77	S	N
83	V	M
84	D	N
93	T	V
115	V	T
116	M	L

### MD simulation of Fabs and key residue identification

**Figure 3:**
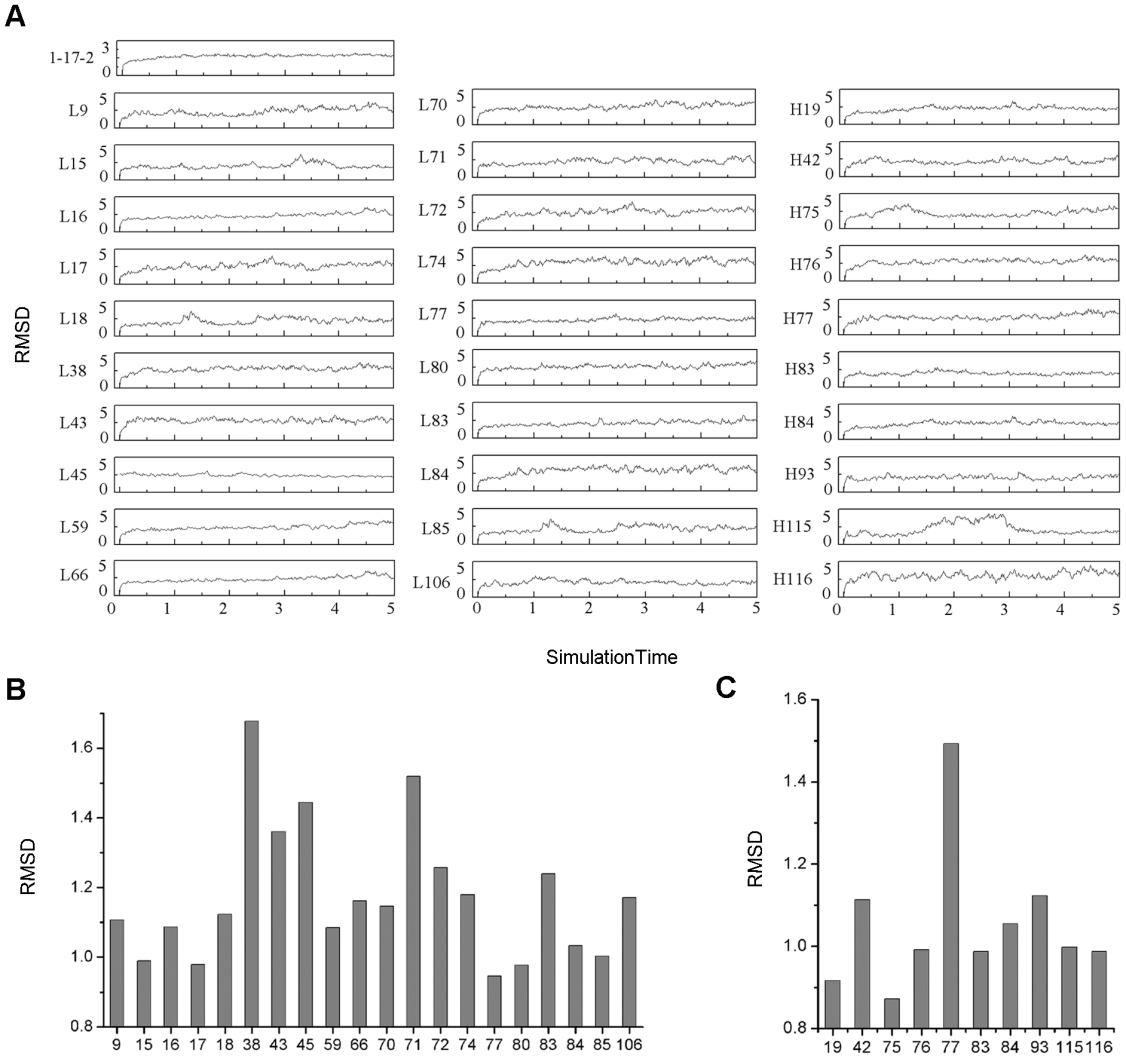
MD simulations and RMSD values of the 30 mutants. (A) The MD simulations of 1-17-2 Fab and the 30 mutants. The curves indicate the RMSD value changes in the MD simulations. (B, C) RMSD value of each mutant that was superimposed with 1-17-2 Fab. The RMSD values of 20 mutants in VL (B) and 10 mutants in VH (C). The RMSDs are calculated by aligning CDRs of mutants and 1-17-2 Fab model.

Corresponding to the 30 substitutions in humanization process, we built 30 mutant Fab models. Virtual mutation was used in building mutant models based on the 1-17-2 Fab model. Although the initial structures had been optimized by EM calculation, which is only a partial minimization that cannot overcome the energy barrier problem, it was necessary to perform a long-range MD simulation to achieve the purpose of the global minimization. The 1-17-2 model and 30 mutant models were performed on 5 ns MD simulation in an explicit water solvent environment. The RMSD curves indicate that most models reached balance when the simulation lasted for 3 ns while some reached balance at 4 ns. The fluctuations in the balance state indicate that the structure of the antibody is flexible rather than static (Fig. 3A). In the end of MD simulation, the thermodynamics average structures were extracted as the final Fab structure.

The RMSD values of the two superimposed proteins reflect the structural differences. The CDRs regions of the mutant structures and 1-17-2 structure were superimposed and RMSD values were calculated. It is generally believed that when the RMSD of two structures are less than 1.0 Å, two structures can be regarded as identical. If the RMSD value of two structures is greater than 1.5 Å, the two structures are different. The RMSD values by 30 mutants are showed in Figure 3B and 3C. Mutants L38, L43, L45, L71 and H77 receive a relatively high RMSD which is close to 1.5 Å. These five amino acids are defined as the key residues in the FRs. In order to improve the affinity of humanized antibody, in the refined antibody, these five key residues were back mutated to rat amino acids from human amino acids.

### Experimental validation of humanized antibody

**Figure 4:**
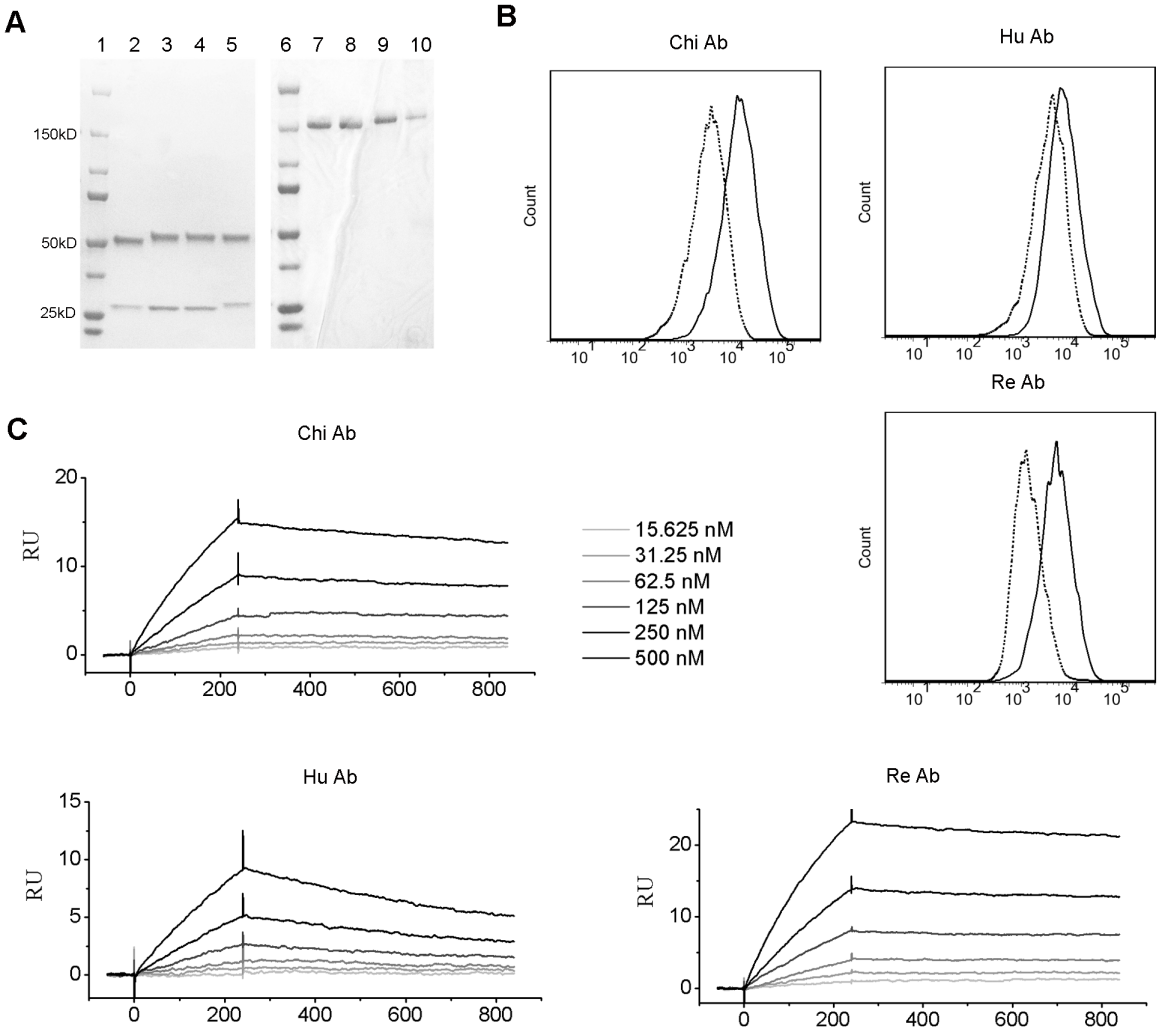
Binding characteristics of the chimeric (Chi), humanized (Hu) and refined (Re) mAbs. (A) SDS gels of the rat, chimeric, humanized and refined mAb under reducing (left) and naïve (right) condition. Lane 2 and 7: rat, lane 3 and 8: chimeric, lane 4 and 9: humanized, lane 5 and 10: refined, lane 1 and 6: marker. The molecular kD was labeled in number. (B) FACS profiles of the chimeric, humanized, and refined 1-17-2 mAb binding to the DEC-1-YB2/0 cells. Dotted lines indicate antibody binding to YB2/0 cells serving as negative controls. FITC labeled anti-human secondary antibody was used to detect surface staining. (C) SPR sensorgrams of the chimeric, humanized, and refined 1-17-2 mAb. Curves of 6 concentrations are shown.

Based on the above analysis and prediction, we constructed chimeric antibody, humanized antibody and refined antibody. We constructed chimeric antibody by combining variable regions of 1-17-2 with constant regions of a human antibody. Humanized antibody was constructed by adding 30 mutations based on the chimeric antibody. Refined antibody was constructed by back mutation of the 5 key residues based on humanized antibody. All the four antibodies were expressed in vitro and purified. The SDS gels under reduced and naïve condition showed that the antibodies were purified and intact (Fig. 4A). Binding characteristics of the three antibodies were compared. Binding ability was first tested by flow cytometric analysis. All three antibodies can bind to DEC-1-YB2/0 cells (Fig. 4B). However, the mean fluorescence intensities (MFI) of the three antibodies were different. Humanized antibody had a smaller MFI than the chimeric antibody. The refined antibody had similar MFI as the chimeric antibody. To directly measure the binding affinity of these mAbs, we used SPR to measure affinity values of these antibodies (Fig. 4C). Humanized antibody had a smaller affinity than the chimeric antibody, indicated by the KD value (Table 3). Refined antibody had a similar affinity as the chimeric antibody (Fig. 4C and Table 3). The decreased affinity of the humanized antibody was caused by a decreased associate rate constant, indicated by a smaller Ka value, and increased dissociate rate constant, indicated by a greater Kd value (Table 3).

**Table 3:** Kinetic constants of Rat, Chimeric, Humanized and Refined mAbs.

	Ka (M-1·S-1)	Kd (S-1)	KD (M)
Chi Ab	8.04±2.07E+03	2.73±0.26E-04	3.54±0.88E-08
Hu Ab	2.93±0.69E+03	1.05±0.11E-03	3.73±1.12E-07
Re Ab	8.22±0.48E+03	1.37±0.06E-04	1.67±0.07E-08
Rat Ab	6.29±0.98E+03	1.85±0.16E-04	2.97±0.22E-08

Ka is the associate rate constant. Kd is the dissociate rate constant. KD is defined by kd/ka ratio. Each constant is the mean and standard deviation of three individual measurements.

### The ability of 1-17-2 mAb to induce internalization in DCs

**Figure 5:**
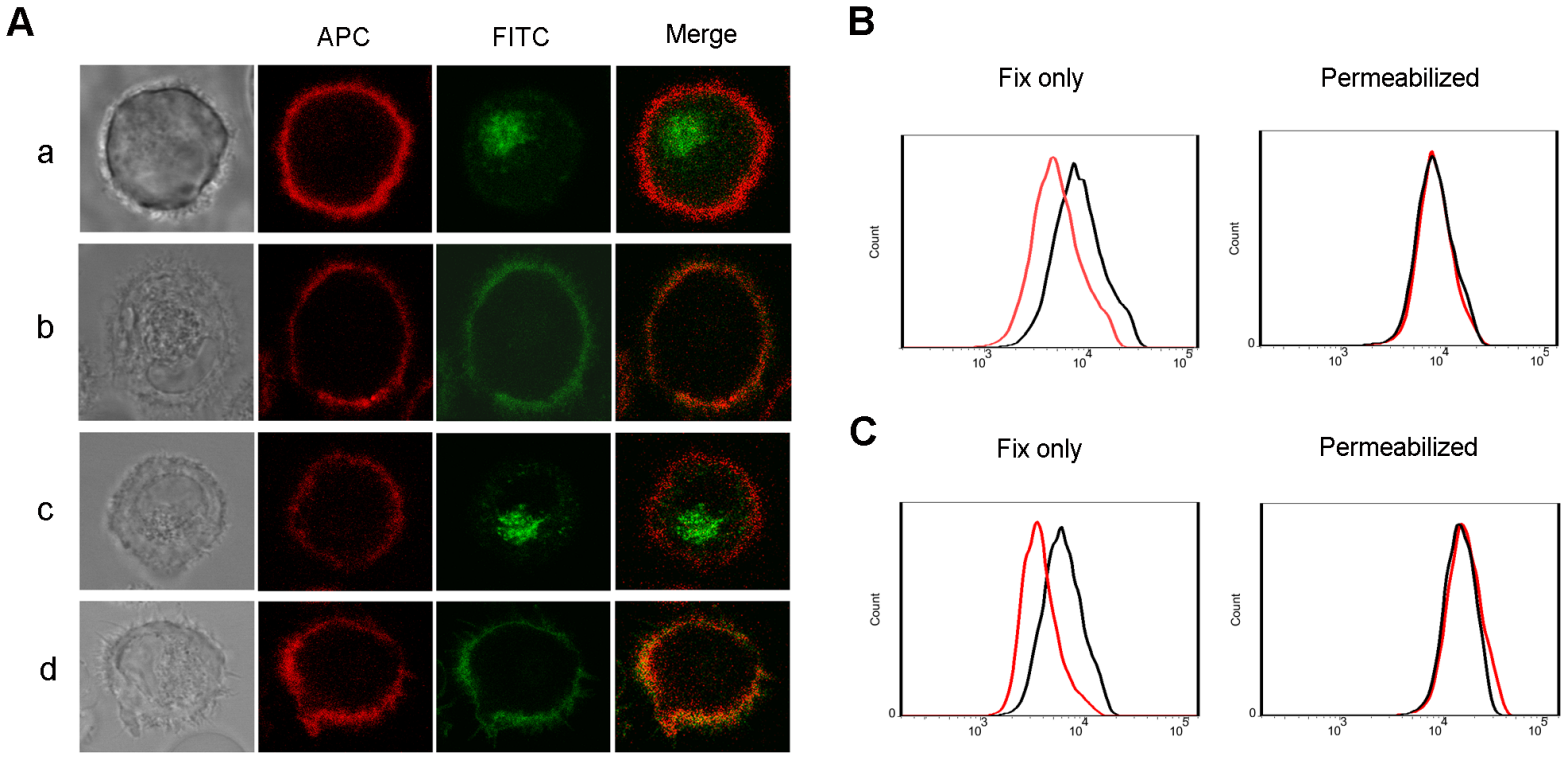
Internalization induced by 1-17-2 rat antibody and refined antibody. (A) Microscopic images of 1-17-2 mAb induced internalization. Cells were stained with APC-labeled anti-CD11c (red), FITC-labeled rat or refined 1-17-2 mAb (green). Bright field images show the shape of cells. Panel a and b: rat 1-17-2 mAb cultured with DCs at 37°C or 4°C respectively. Panel c and d: refined 1-17-2 mAb cultured with DCs at 37°C and 4°C respectively. (B, C) Staining levels of rat (B) and refined (C) 1-17-2 mAb on DCs. Fix only indicates detection of mAb staining on the cell surface. Permeabilized indicates detection of mAb staining on the surface and intracellularlly. Red line: DCs incubated at 37°C; Black line: DCs incubated at 4°C.

The major function of anti-DEC-205 antibody is to induce the internalization of DEC-205 antigen/antibody complex in DCs. We then examined the internalization of 1-17-2 rat antibody and the refined antibody by DCs. We cultured DCs with FITC-labeled rat or refined antibodies at 37°C or 4°C and observed fluorescence distribution under laser scanning microscope. As shown in Figure 5A, the majority of the FITC fluorescence appeared inside the cell membrane boundary (panel a and c) when cultured at 37°C. In contrast, the green fluorescence appeared on the cell membrane and was overlapped with red fluorescence when cells were cultured at 4°C (Fig. 5A, panel b and d), indicating that the labeled antibodies were endocytosed. To further determine antibody endocytosis, we incubated DCs with antibodies at 37°C and used flow cytometry assay to detect the internalization of antibodies by DCs. Binding of FITC anti-human/rat antibody to fixed cells measures surface antibody, and binding to permeabilized cells measures both surface and intracellular antibody. As showed in Figure 5B and 5C, surface antibody levels were decreased after incubating at 37°C for 1h, suggesting that the refined antibody has a similar ability to that of 1-17-2 to induce internalization.

## Discussion

The ultimate goal of antibody humanization is to substitute murine residues with the human counterparts while keep its binding affinity to the maximum extent. The dilemma is that more humanizing substitutions likely reduce more the antibody’s binding affinity. Sometimes certain degree of binding affinity is compromised to achieve humanization, or antibody is not fully humanized in order to retain its binding affinity. The binding affinity of antibody depends on the conformation of the six CDRs that are supported by the FRs within the variable domain [45]. Humanizing substitutions in the FRs usually change the conformation of CDRs thus reducing the binding affinity. Traditional humanization methods are mostly based on sequence alignment and static modeling. These methods are difficult to accurately acquire the dynamic CDRs conformation that is most important in assessing the relationship between antibody affinity and residues substitution.

Chimeric antibody usually has the same binding affinity with the original murine antibody [11], [46]. Our data also confirm that the change of binding affinity of the 1-17-2 chimeric antibody is minimal. Traditional humanization method of CDR-grafting chooses only one single human antibody FR as template by similarity alignment [17], [47]. As there is diversity in FRs among different human antibodies, we hypothesized that the immunogenicity of rat antibody FR can be removed by epitope scanning in multiple human templates. By only swapping out the antigenic residues, the FR can keep much more harmless rat residues that are important in maintaining the antibody binding affinity.

The RMSD calculation identified five key residues in antibody 1-17-2. They are position 38, 43, 45, and 71 in light chain and position 77 in heavy chain. After back mutation, the binding affinity of the antibody was rescued to the level of chimera antibody. Further analysis showed that residues 38 in the light chain are located on the interface between VL and VH domain. Mutation from Leu to Gln generates a big difference in side chain. Moreover, Gln can form hydrogen bonds with the heavy chain. The same situation occurs in residue 43 of the light chain, mutating from Ser to Ala. The positions in 45 and 71 of the light chain and 77 of the heavy chain are located in the border of the CDRs that could affect the CDRs directly. Mutations on these sites all have a change in the side chain. Thus, residues on the interface between the VL and VH domain and residues on the borders of CDRs are pivotal.

Computer-aided analysis methods are playing an increasingly important role in modern biological research [23], [26], [48]. In this study virtual mutation and MD simulation were combined to discover the structural impact of some amino acid mutation on CDRs. This combination can reduce the blindness of experiment and the time consumption. Moreover MD simulation as an important molecular modeling [49] method is the core technology in this work to solve the problem of amino acids change and its structural impact on CDRs. The MD calculation often requires high-performance computer. We used a massively parallel MD software Gromacs and let it run in the high-performance computing cluster, making the explicit solvent water MD reach 5ns. Our results show that for only a few amino acid mutations 5ns is sufficient for searching the most reasonable conformation.

For the final structures obtained from MD, we calculated the RMSD to reflect the impact of mutation on the CDRs conformation. The size of the RMSD values reflects the degree of difference between the two structures. In traditional conformational analysis, when the RMSD of two structures is greater than 2.0 Å, the two structures are considered to be significantly different. In our work here, RMSDs greater than 2.0 Å were not found. Two possible reasons may explain this. First, the present study was conducted in a single amino acid mutation, while the actual conformation change is caused by the combination of a plurality of amino acids mutation. Conformational change by a single point mutation is limited. Second, the interaction of antigen and antibody is strictly spatial conformation dependent and extremely sensitive to any slight change. RMSD values greater than 1.5 Å may also cause a significant decrease of binding affinity, so we take RMSD value 1.5 Å as a threshold to determine the key residues.

By epitope scanning, we identified 30 epitopic residues in the FRs. After substitution with human amino acids, we obtained the humanized antibody with only CDR regions of rat origin. The humanization rate is 90.5% (calculated by the amino acid number of FRs 600 divided by total amino acid number 663). Then, we generalized refined antibody with back-mutation of 5 amino acids in FRs. The humanization rate was reduced to 89.7% (595 divided by 663). The refined antibody has a great improvement in binding affinity, with a slightly decreased humanization rate, which we think is the necessary compromise. We further used in silico method to predict whether the back-mutation site in the refined antibody causes new epitope. We used IEDB web server to analyze the MHC II binding of the peptide contain the back-mutation sites [50], [51]. The peptides with back-mutation didn’t have high affinity with the eight MHC II alleles, which represent almost 90% of MHC diversity [52], as show in Table S1. The result showed that the refined antibody doesn’t cause additional MHC II epitopes.

In summary, we have developed a novel antibody humanization method. Based on the Fab structure prediction, a novel epitopes scanning algorithm was designed to identify all the mutants. Then virtual mutation and MD simulation that form the core of this work were performed to discover key residues in the FRs that may have great structural impact on CDRs. Experiment results validated our prediction. The antibody with back mutations exhibits similar binding affinity compared with the chimera antibody. Moreover, antibody 1-17-2 showed a great ability to induce internalization on DCs. These results indicate a promising therapeutic prospective.

## Supporting Information

Table S1 **Peptide affinity with 8 MHC alleles.**
(DOC)

## References

[1] Swann PG, Tolnay M, Muthukkumar S, Shapiro MA, Rellahan BL, et al (2008) Considerations for the development of therapeutic monoclonal antibodies. Curr Opin Immunol 20: 493–499. 18586093 10.1016/j.coi.2008.05.013

[2] Reichert JM (2012) Marketed therapeutic antibodies compendium. MAbs 4: 413–415. 22531442 10.4161/mabs.19931 PMC3355480

[3] Jiang W, Swiggard WJ, Heufler C, Peng M, Mirza A, et al (1995) The receptor DEC-205 expressed by dendritic cells and thymic epithelial cells is involved in antigen processing. Nature 375: 151–155. 7753172 10.1038/375151a0

[4] Figdor CG, van Kooyk Y, Adema GJ (2002) C-type lectin receptors on dendritic cells and Langerhans cells. Nat Rev Immunol 2: 77–84. 11910898 10.1038/nri723

[5] Lahoud MH AF, Zhang JG, Meuter S, Policheni AN, Kitsoulis S, et al (2012) DEC-205 is a cell surface receptor for CpG oligonucleotides. Proc Natl Acad Sci U S A 109: 16270–16275. 22988114 10.1073/pnas.1208796109 PMC3479608

[6] Bonifaz LC, Bonnyay DP, Charalambous A, Darguste DI, Fujii S, et al (2004) In vivo targeting of antigens to maturing dendritic cells via the DEC-205 receptor improves T cell vaccination. J Exp Med 199: 815–824. 15024047 10.1084/jem.20032220 PMC2212731

[7] Presta LG (2006) Engineering of therapeutic antibodies to minimize immunogenicity and optimize function. Adv Drug Deliv Rev 58: 640–656. 16904789 10.1016/j.addr.2006.01.026

[8] Nelson AL, Dhimolea E, Reichert JM (2010) Development trends for human monoclonal antibody therapeutics. Nat Rev Drug Discov 9: 767–774. 20811384 10.1038/nrd3229

[9] Calvo B, Zuniga L (2012) Therapeutic monoclonal antibodies: strategies and challenges for biosimilars development. Curr Med Chem 19: 4445–4450. 22978327 10.2174/092986712803251485

[10] Almagro JC, Fransson J (2008) Humanization of antibodies. Front Biosci 13: 1619–1633. 17981654 10.2741/2786

[11] Hackett J Jr, Hoff-Velk J, Golden A, Brashear J, Robinson J, et al (1998) Recombinant mouse-human chimeric antibodies as calibrators in immunoassays that measure antibodies to Toxoplasma gondii. J Clin Microbiol 36: 1277–1284. 9574691 10.1128/jcm.36.5.1277-1284.1998 PMC104814

[12] Tjandra JJ, Ramadi L, McKenzie IF (1990) Development of human anti-murine antibody (HAMA) response in patients. Immunol Cell Biol 68 ( Pt 6): 367–376. 10.1038/icb.1990.50 1711007

[13] Jones PT, Dear PH, Foote J, Neuberger MS, Winter G (1986) Replacing the complementarity-determining regions in a human antibody with those from a mouse. Nature 321: 522–525. 3713831 10.1038/321522a0

[14] Verhoeyen M, Milstein C, Winter G (1988) Reshaping human antibodies: grafting an antilysozyme activity. Science 239: 1534–1536. 2451287 10.1126/science.2451287

[15] Kennedy GA, Tey SK, Cobcroft R, Marlton P, Cull G, et al (2002) Incidence and nature of CD20-negative relapses following rituximab therapy in aggressive B-cell non-Hodgkin's lymphoma: a retrospective review. Br J Haematol 119: 412–416. 12406079 10.1046/j.1365-2141.2002.03843.x

[16] Lazar GA, Desjarlais JR, Jacinto J, Karki S, Hammond PW (2007) A molecular immunology approach to antibody humanization and functional optimization. Mol Immunol 44: 1986–1998. 17079018 10.1016/j.molimm.2006.09.029

[17] Chaudhary VK, Queen C, Junghans RP, Waldmann TA, FitzGerald DJ, et al (1989) A recombinant immunotoxin consisting of two antibody variable domains fused to Pseudomonas exotoxin. Nature 339: 394–397. 2498664 10.1038/339394a0

[18] Kettleborough CA, Saldanha J, Heath VJ, Morrison CJ, Bendig MM (1991) Humanization of a mouse monoclonal antibody by CDR-grafting: the importance of framework residues on loop conformation. Protein Eng 4: 773–783. 1798701 10.1093/protein/4.7.773

[19] Padlan EA (1991) A possible procedure for reducing the immunogenicity of antibody variable domains while preserving their ligand-binding properties. Mol Immunol 28: 489–498. 1905784 10.1016/0161-5890(91)90163-e

[20] Pedersen JT, Henry AH, Searle SJ, Guild BC, Roguska M, et al (1994) Comparison of surface accessible residues in human and murine immunoglobulin Fv domains. Implication for humanization of murine antibodies. J Mol Biol 235: 959–973. 7507176 10.1006/jmbi.1994.1050

[21] Roguska MA, Pedersen JT, Henry AH, Searle SM, Roja CM, et al (1996) A comparison of two murine monoclonal antibodies humanized by CDR-grafting and variable domain resurfacing. Protein Eng 9: 895–904. 8931129 10.1093/protein/9.10.895

[22] Sivasubramanian A, Sircar A, Chaudhury S, Gray JJ (2009) Toward high-resolution homology modeling of antibody Fv regions and application to antibody-antigen docking. Proteins 74: 497–514. 19062174 10.1002/prot.22309 PMC2909601

[23] Caravella JA, Wang D, Glaser SM, Lugovskoy A (2010) Structure-Guided Design of Antibodies. Curr Comput Aided Drug Des. 26845329

[24] Vyas VK, Ukawala RD, Ghate M, Chintha C (2012) Homology modeling a fast tool for drug discovery: current perspectives. Indian J Pharm Sci 74: 1–17. 23204616 10.4103/0250-474X.102537 PMC3507339

[25] Mo SL, Liu WF, Li CG, Zhou ZW, Luo HB, et al (2012) Pharmacophore, QSAR, and binding mode studies of substrates of human cytochrome P450 2D6 (CYP2D6) using molecular docking and virtual mutations and an application to chinese herbal medicine screening. Curr Pharm Biotechnol 13: 1640–1704. 22039821 10.2174/138920112800958779

[26] Cheng X, Ivanov I (2012) Molecular dynamics. Methods Mol Biol 929: 243–285. 23007433 10.1007/978-1-62703-050-2_11

[27] Coutsias EA, Seok C, Dill KA (2004) Using quaternions to calculate RMSD. J Comput Chem 25: 1849–1857. 15376254 10.1002/jcc.20110

[28] Lefranc MP, Pommie C, Ruiz M, Giudicelli V, Foulquier E, et al (2003) IMGT unique numbering for immunoglobulin and T cell receptor variable domains and Ig superfamily V-like domains. Dev Comp Immunol 27: 55–77. 12477501 10.1016/s0145-305x(02)00039-3

[29] Yuan XH, Qu ZY, Wu XM, Wang YC, Wei FX, et al (2009) Homology Modeling and Evolution Trace Analysis of Human Adenovirus Type 3 Hexon. Chem J Chinese Universities 30: 1636.

[30] Eswar N, Webb B, Marti-Renom MA, Madhusudhan MS, Eramian D, et al.. (2007) Comparative protein structure modeling using MODELLER. Curr Protoc Protein Sci Chapter 2: Unit 2 9. 10.1002/0471140864.ps0209s50 18429317

[31] Spassov VZ, Flook PK, Yan L (2008) LOOPER: a molecular mechanics-based algorithm for protein loop prediction. Protein Eng Des Sel 21: 91–100. 18194981 10.1093/protein/gzm083

[32] MacKerell AD Jr, Banavali N, Foloppe N (2000) Development and current status of the CHARMM force field for nucleic acids. Biopolymers 56: 257–265. 11754339 10.1002/1097-0282(2000)56:4<257::AID-BIP10029>3.0.CO;2-W

[33] Yuan XH, WANG Y-C, QU ZY, REN JY, WANG JF, et al (2011) Novel Rapid Molecular Modeling Method Based on Evolutional Tree for Human Adenovirus Hexon Proteins Family. Chem J Chinese Universities 32: 1838.

[34] Yuan XH, Wang YC, Jin WJ, Zhao BB, Chen CF, et al (2012) Structure-based high-throughput epitope analysis of hexon proteins in B and C species human adenoviruses (HAdVs). PLoS One 7: e32938. 22427913 10.1371/journal.pone.0032938 PMC3302796

[35] Laskowski RA, Rullmannn JA, MacArthur MW, Kaptein R, Thornton JM (1996) AQUA and PROCHECK-NMR: programs for checking the quality of protein structures solved by NMR. J Biomol NMR 8: 477–486. 9008363 10.1007/BF00228148

[36] Suyama M, Matsuo Y, Nishikawa K (1997) Comparison of protein structures using 3D profile alignment. J Mol Evol 44 Suppl 1S163–173. 9071025 10.1007/pl00000065

[37] Larkin MA, Blackshields G, Brown NP, Chenna R, McGettigan PA, et al (2007) Clustal W and Clustal X version 2.0. Bioinformatics 23: 2947–2948. 17846036 10.1093/bioinformatics/btm404

[38] Yuan X, Qu Z, Wu X, Wang Y, Liu L, et al (2009) Molecular modeling and epitopes mapping of human adenovirus type 3 hexon protein. Vaccine 27: 5103–5110. 19573641 10.1016/j.vaccine.2009.06.041

[39] Van Der Spoel D, Lindahl E, Hess B, Groenhof G, Mark AE, et al (2005) GROMACS: fast, flexible, and free. J Comput Chem 26: 1701–1718. 16211538 10.1002/jcc.20291

[40] Chatterjee S, Debenedetti PG, Stillinger FH, Lynden-Bell RM (2008) A computational investigation of thermodynamics, structure, dynamics and solvation behavior in modified water models. J Chem Phys 128: 124511. 18376947 10.1063/1.2841127

[41] Cheatham TE, Miller JL, Fox T, Darden TA, Kollman PA (1995) Molecular Dynamics Simulations on Solvated Biomolecular Systems: The Particle Mesh Ewald Method Leads to Stable Trajectories of DNA, RNA, and Proteins. J Am Chem Soc 117: 4193.

[42] Coutsias EA, Seok C, Dill KA (2004) Using quaternions to calculate RMSD. J Comput Chem 25: 1849. 15376254 10.1002/jcc.20110

[43] Zhou LJ, Tedder TF (1996) CD14+ blood monocytes can differentiate into functionally mature CD83+ dendritic cells. Proc Natl Acad Sci U S A 93: 2588–2592. 8637918 10.1073/pnas.93.6.2588 PMC39841

[44] Brady JL, Corbett AJ, McKenzie BS, Lew AM (2006) Rapid specific amplification of rat antibody cDNA from nine hybridomas in the presence of myeloma light chains. J Immunol Methods 315: 61–67. 16901500 10.1016/j.jim.2006.07.002

[45] Poljak RJ, Amzel LM, Chen BL, Phizackerley RP, Saul F (1974) The three-dimensional structure of the fab' fragment of a human myeloma immunoglobulin at 2.0-angstrom resolution. Proc Natl Acad Sci U S A 71: 3440–3444. 4215080 10.1073/pnas.71.9.3440 PMC433789

[46] Morrison SL, Johnson MJ, Herzenberg LA, Oi VT (1984) Chimeric human antibody molecules: mouse antigen-binding domains with human constant region domains. Proc Natl Acad Sci U S A 81: 6851–6855. 6436822 10.1073/pnas.81.21.6851 PMC392030

[47] Carter P, Presta L, Gorman CM, Ridgway JB, Henner D, et al (1992) Humanization of an anti-p185HER2 antibody for human cancer therapy. Proc Natl Acad Sci U S A 89: 4285–4289. 1350088 10.1073/pnas.89.10.4285 PMC49066

[48] Nurisso A, Daina A, Walker RC (2012) A practical introduction to molecular dynamics simulations: applications to homology modeling. Methods Mol Biol 857: 137–173. 22323220 10.1007/978-1-61779-588-6_6

[49] Friedman R, Boye K, Flatmark K (2013) Molecular modelling and simulations in cancer research. Biochim Biophys Acta. 10.1016/j.bbcan.2013.02.001 23416097

[50] Wang P, Sidney J, Dow C, Mothe B, Sette A, et al (2008) A systematic assessment of MHC class II peptide binding predictions and evaluation of a consensus approach. . PloS Comput Biol. 4(4): e1000048. 18389056 10.1371/journal.pcbi.1000048 PMC2267221

[51] Wang P, Sidney J, Kim Y, Sette A, Lund O, et al (2010) Peptide binding predictions for HLA DR, DP and DQ molecules. BMC Bioinformatics. 11: 568. 21092157 10.1186/1471-2105-11-568 PMC2998531

[52] Southwood S, Sidney J, Kondo A, Guercio del MF, Appella E, et al (1998) Several common HLA-DR types share largely overlapping peptide binding repertoires. . J. Immunol. 160: 3363–3373. 9531296

