# Supplementary material for: A Novel Antibody Humanization Method Based on Epitopes Scanning and Molecular Dynamics Simulation
Source: PLoS One. 2013 Nov 21;8(11):e80636. doi: 10.1371/journal.pone.0080636 (PMC3836750; doi:10.1371/journal.pone.0080636)
Supplement: Table S1 — Peptide affinity with 8 MHC alleles. (DOC) [file pone.0080636.s001.doc]

TABLE S1. Peptide affinity with 8 MHC alleles.

| Sequence | Allele | Method used | Percentile Rank |
| --- | --- | --- | --- |
| ISRDNSKNTLYLQMN | HLA-DRB1*0101 | Consensus (comb.lib.,smm,nn) | 74.16 |
|  | HLA-DRB1*0301 | Consensus (smm,nn,sturniolo) | 12.14 |
|  | HLA-DRB1*0401 | Consensus (smm,nn,sturniolo) | 10.39 |
|  | HLA-DRB1*0701 | Consensus (comb.lib.,smm,nn) | 66.66 |
|  | HLA-DRB1*0801 | sturniolo | 28.22 |
|  | HLA-DRB1*1101 | Consensus (smm,nn,sturniolo) | 29.78 |
|  | HLA-DRB1*1301 | sturniolo | 27.33 |
|  | HLA-DRB1*1501 | Consensus (smm,nn,sturniolo) | 32.83 |
| ISRDNSKSTLYLQMN | HLA-DRB1*0101 | Consensus (comb.lib.,smm,nn) | 67.55 |
|  | HLA-DRB1*0301 | Consensus (smm,nn,sturniolo) | 12.14 |
|  | HLA-DRB1*0401 | Consensus (smm,nn,sturniolo) | 9.11 |
|  | HLA-DRB1*0701 | Consensus (comb.lib.,smm,nn) | 43.62 |
|  | HLA-DRB1*0801 | sturniolo | 28.22 |
|  | HLA-DRB1*1101 | Consensus (smm,nn,sturniolo) | 29.78 |
|  | HLA-DRB1*1301 | sturniolo | 27.33 |
|  | HLA-DRB1*1501 | Consensus (smm,nn,sturniolo) | 33.88 |
| WYQQKPGKAPKLLIY | HLA-DRB1*0101 | Consensus (comb.lib.,smm,nn) | 39.64 |
|  | HLA-DRB1*0301 | Consensus (smm,nn,sturniolo) | 16.6 |
|  | HLA-DRB1*0401 | Consensus (smm,nn,sturniolo) | 29.55 |
|  | HLA-DRB1*0701 | Consensus (comb.lib.,smm,nn) | 52.05 |
|  | HLA-DRB1*0801 | sturniolo | 6.39 |
|  | HLA-DRB1*1101 | Consensus (smm,nn,sturniolo) | 6.63 |
|  | HLA-DRB1*1301 | sturniolo | 17.36 |
|  | HLA-DRB1*1501 | Consensus (smm,nn,sturniolo) | 37.96 |
| WYQLKPGKSPQLLIY | HLA-DRB1*0101 | Consensus (comb.lib.,smm,nn) | 13.55 |
|  | HLA-DRB1*0301 | Consensus (smm,nn,sturniolo) | 9.93 |
|  | HLA-DRB1*0401 | Consensus (smm,nn,sturniolo) | 6.96 |
|  | HLA-DRB1*0701 | Consensus (comb.lib.,smm,nn) | 17.75 |
|  | HLA-DRB1*0801 | sturniolo | 2.37 |
|  | HLA-DRB1*1101 | Consensus (smm,nn,sturniolo) | 1.08 |
|  | HLA-DRB1*1301 | sturniolo | 10.35 |
|  | HLA-DRB1*1501 | Consensus (smm,nn,sturniolo) | 12.67 |
| GSGSGTDFTLTISSL | HLA-DRB1*0101 | Consensus (comb.lib.,smm,nn) | 61.31 |
|  | HLA-DRB1*0301 | Consensus (smm,nn,sturniolo) | 34.99 |
|  | HLA-DRB1*0401 | Consensus (smm,nn,sturniolo) | 34.99 |
|  | HLA-DRB1*0701 | Consensus (comb.lib.,smm,nn) | 19.29 |
|  | HLA-DRB1*0801 | sturniolo | 34.99 |
|  | HLA-DRB1*1101 | Consensus (smm,nn,sturniolo) | 34.99 |
|  | HLA-DRB1*1301 | sturniolo | 34.99 |
|  | HLA-DRB1*1501 | Consensus (smm,nn,sturniolo) | 71.1 |
| GSGSGTDYTLTISSL | HLA-DRB1*0101 | Consensus (comb.lib.,smm,nn) | 52.59 |
|  | HLA-DRB1*0301 | Consensus (smm,nn,sturniolo) | 34.99 |
|  | HLA-DRB1*0401 | Consensus (smm,nn,sturniolo) | 34.99 |
|  | HLA-DRB1*0701 | Consensus (comb.lib.,smm,nn) | 16 |
|  | HLA-DRB1*0801 | sturniolo | 34.99 |
|  | HLA-DRB1*1101 | Consensus (smm,nn,sturniolo) | 34.99 |
|  | HLA-DRB1*1301 | sturniolo | 34.99 |
|  | HLA-DRB1*1501 | Consensus (smm,nn,sturniolo) | 68.86 |

ISRDNSKNTLYLQMN is unique from humanized heavy chain; ISRDNSKSTLYLQMN from refined heavy chain; WYQQKPGKAPKLLIY and GSGSGTDFTLTISSL from humanized light chain; WYQLKPGKSPQLLIY and GSGSGTDYTLTISSL from refined light chain. Percentile rank represents affinity between peptide and MHC allele, a small number indicates high affinity. Usually, percentile rank smaller than 1 indicates an epitope. The IEDB web server use Consensus method to predict the binding with HLA-DRB*0101, HLA-DRB*0301, HLA-DRB*0401, HLA-DRB*0701, HLA-DRB*1101, HLA-DRB*1501, and use sturniolo method to predict the binding with HLA-DRB*0801, HLA-DRB*1301.
